# A 33.5 cm Mucinous Ovarian Carcinoma and Associated Comorbidities in an 89-Year-Old Female

**DOI:** 10.7759/cureus.67970

**Published:** 2024-08-27

**Authors:** Hadia A Tariq, Fahad I Abid, Joseph Galloway

**Affiliations:** 1 Department of Research, Alabama College of Osteopathic Medicine, Dothan, USA; 2 Department of Surgery, Mobile Infirmary Medical Center, Mobile, USA

**Keywords:** mucinous ovarian carcinoma, tumor, mucin, carcinoma, ovarian cancer

## Abstract

Ovarian cancer is known to cause the highest mortality of gynecologic cancers. We present the case of an 89-year-old Caucasian female who presented to her surgeon with a large 33.5 cm abdominopelvic mass. Imaging done before surgery and surgical resection found the mass to be a very large ovarian tumor, and subsequent histopathology found the tumor to be a moderately differentiated mucinous ovarian carcinoma (MOC) that weighed 8.16 kg with dimensions of 33.5 × 32.1 × 16.5 cm. Histological staining revealed the tumor cells to be nonspecific overall but in favor of a primarily ovarian source. While primary MOCs tend to be relatively larger when compared to other ovarian tumors (>10 cm), there have been few documented cases of ovarian tumors, specifically primary MOCs, presenting at this large of a size.

## Introduction

Out of all gynecologic cancers, ovarian cancer is known to cause the highest mortality [1]. This is likely due to the lack of reliable routine screening tests and the overall asymptomatic course of most tumors. While the majority of ovarian tumors tend to be benign, malignant tumors when present, are a leading cause of death. Rare subtypes such as mucinous ovarian carcinoma (MOC) account for roughly 2%-3% of newly diagnosed cases. Mucinous neoplasms of the ovary typically affect women between the ages of 20 and 40, and while their clinical presentation may be nonspecific, they typically present as a large unilateral pelvic mass [2].

MOC patients may present with complaints of fatigue, bloating, abdominopelvic pain, or discomfort, likely due to mass effect [3]. Patients with advanced MOC exhibit poorer survival rates, possibly attributed to the overall aggressive nature of MOC, its tendency to metastasize, or its resistance to traditional platinum-based therapy, an approach that has been proven to be effective for other epithelial ovarian carcinomas (EOCs) [4]. A distinct feature of MOC is it may resemble gastrointestinal (GI) tract tissue, which poses an added challenge in identifying the tumor's origin as either ovary or metastatic cancer [3].

## Case presentation

**Figure 1:**
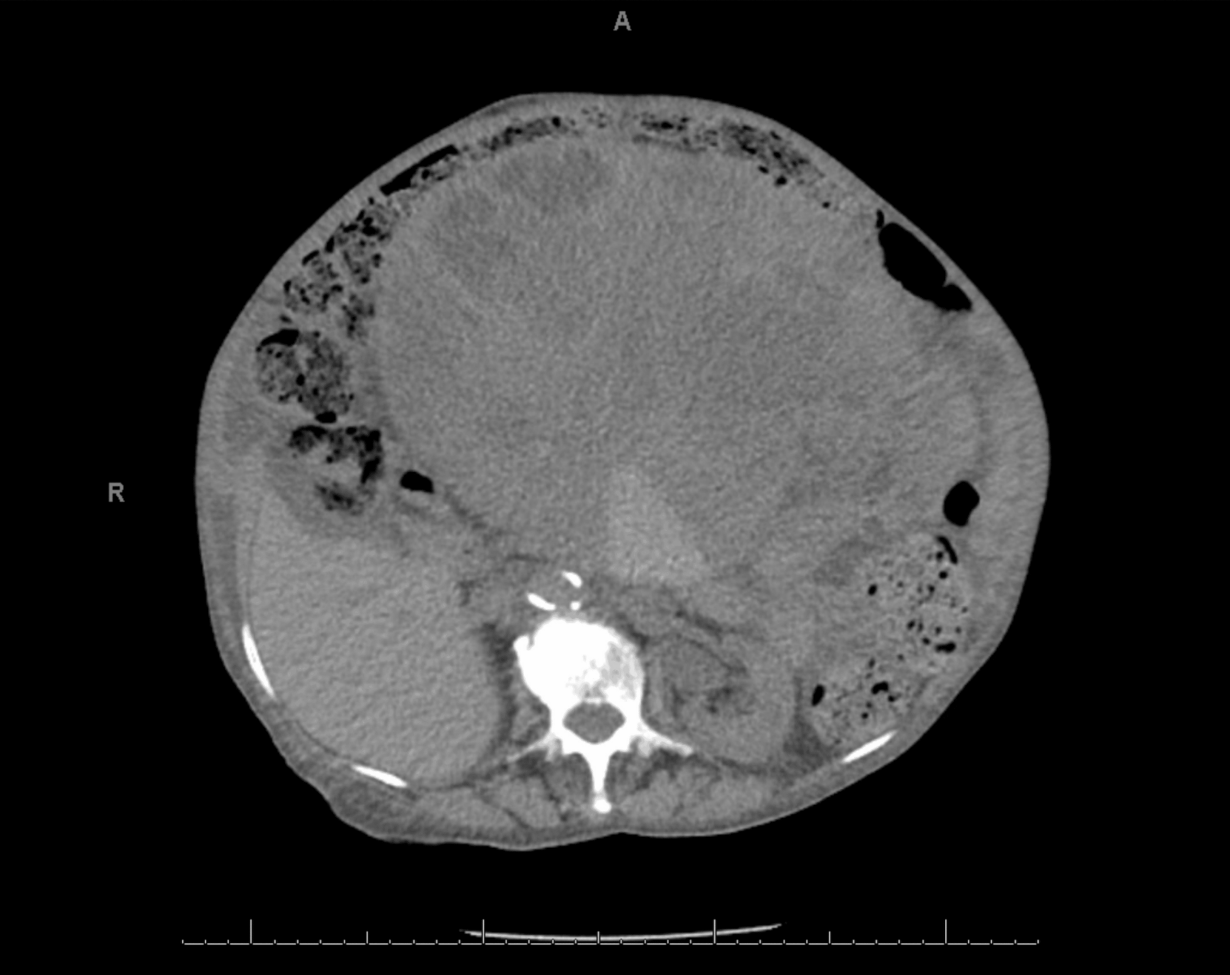
A transverse abdominal CT scan of the abdomen and pelvis without IV contrast reveals a large abdominopelvic mass (measuring 33.5 cm) occupying a significant portion of the abdominal cavity, displacing adjacent structures. The mass appears heterogeneous in density, suggesting the possibility of a complex cystic or solid lesion, which was consistent with specimen findings after surgical removal. CT: computed tomography, IV: intravenous

**Figure 2:**
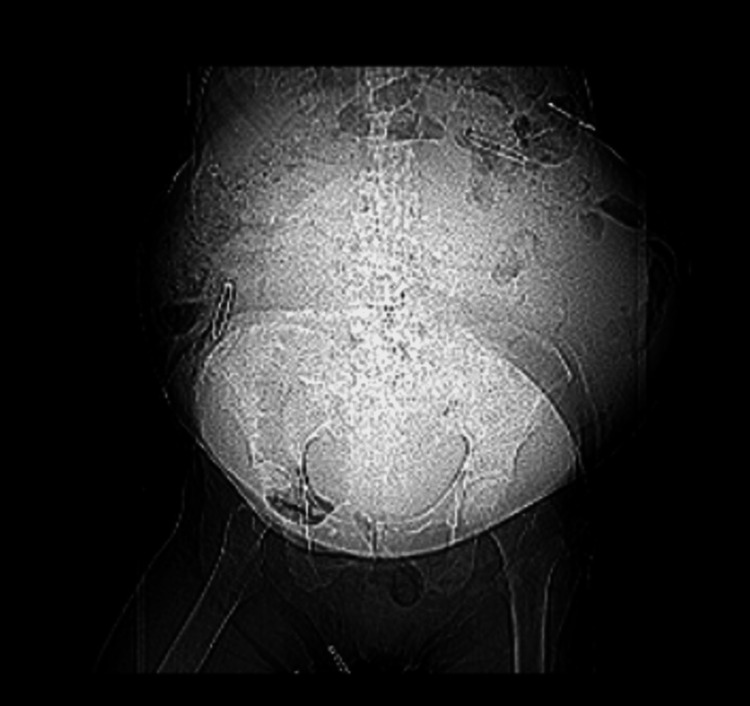
A coronal abdominal CT scan without IV contrast shows the 33.5 cm mass causing significant abdominal distention, correlating with the patient's clinical picture. CT: computed tomography, IV: intravenous

An 89-year-old Caucasian female presented to her gynecologic oncologist with complaints of a large abdominal mass and associated symptoms of abdominal distention, gastroesophageal reflux, dyspnea, and bilateral lower extremity swelling. She had a past medical and surgical history of a 17 cm ovarian cellular fibroma and hysterectomy in 2011, ventral hernia repair with mesh, and hypothyroidism. A computed tomography (CT) scan of the abdomen and pelvis without IV contrast revealed a 33.5 cm abdominopelvic mass, a very large ventral incisional hernia, and a hiatal hernia with gastric and pancreatic components (Figure 1 and Figure 2). A lower extremity venous duplex scan found bilateral femoral deep vein thromboses (DVTs) in her right leg with profuse bilateral lower extremity swelling likely caused by inferior vena cava (IVC) compression due to the mass. A joint procedure between gynecologic oncology and general surgery for an open hiatal hernia and ventral herniorrhaphy with exploratory laparotomy for pelvic mass resection along with bilateral salpingectomy and oophorectomy was performed.

**Figure 3:**
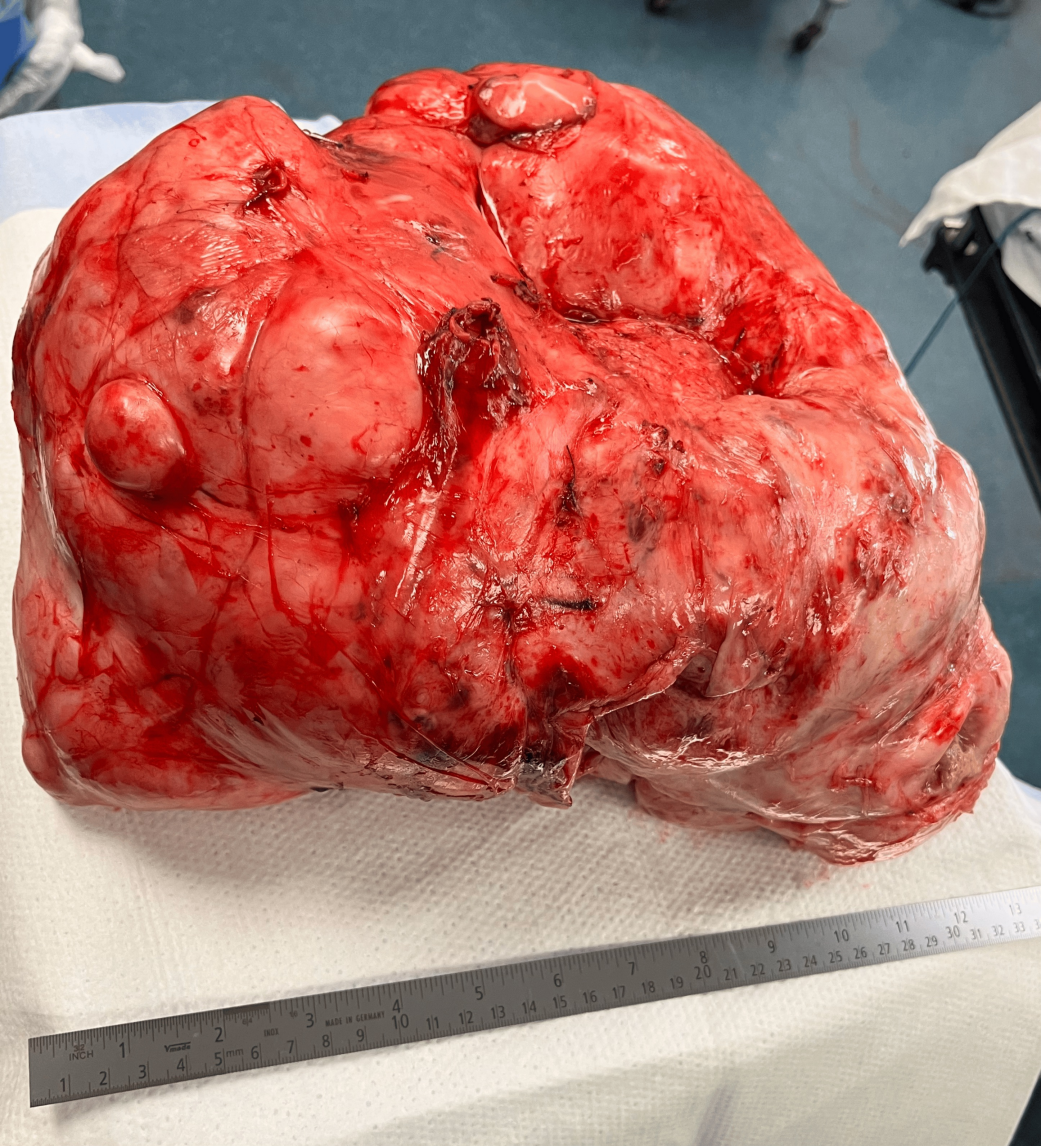
Specimen showing a 33.5 × 32.1 × 16.5 cm mucinous ovarian carcinoma weighing 8.16 kg. The surface of the mass is multiloculated with mucin-containing cysts.

Prior to surgery, she underwent a prophylactic IVC filter placement to address the risk of a clot embolus due to IVC decompression with plans for postoperative anticoagulant therapy. On the day of surgery, initial findings showed the transverse colon stretched across the ventral surface of the mass. Using careful and tedious blunt dissection, we were able to separate the colon from the mass and enter the peritoneal cavity. A combination of sharp and blunt dissection was used to lyse adhesions to separate the transverse colon and sigmoid colon from the anterior and left lateral surface of the mass and cauterize the vascular attachments. The mass was elevated from the abdominal cavity to visualize the posterior surface and lyse adhesions to the underlying bowel mesentery. Roughly 1,500 ccs of fluid was drained from the mass, which was then removed and sent to pathology (Figure 3). A left adnexal mass, possibly an ovary, was also resected and sent for pathology. After the stomach was reduced from the hiatal hernia, the intra-abdominal structures were able to return to the abdomen from the chest and left upper quadrant after mass removal. The abdomen was irrigated, and a 19 French round JP drain was placed in the pelvis and brought out the right lower quadrant stab incision. The surgery concluded with the surgical incision being closed and cleaned, and the patient being transferred to the surgical intensive care unit. The surgical time was approximately two hours, and the estimated blood loss was 1,200 mL. The patient's postoperative course was uneventful. The patient was advised to undergo close outpatient follow-up with her gynecologic oncologist as the specimen pathology report denoted clear negative margins.

Pathology findings reported the mass to be a moderately differentiated mucinous ovarian carcinoma with extension into adjacent smooth muscle and abdominal wall; however, the margins of resection were negative and well visualized. The tan-red nodular mass weighed 8.16 kg with the following dimensions: 33.5 × 32.1 × 16.5 cm. There were multiple translucent, softened nodules containing clear, thin fluid measuring up to 7.5 cm in greatest dimension. Under the International Federation of Gynecology and Obstetrics (FIGO) staging criteria, the specimen was identified as stage IIb. Histological staining revealed that the tumor cells were positive for CDX2, patchy positive for CK7, and negative for CK20, PAX8, WT1, and calretinin. Although the staining pattern was nonspecific, an ovarian primary was favored based on the overall histological and immunohistochemical findings; however, a gastrointestinal primary could not be entirely excluded.

## Discussion

While ovarian cancer is one of the leading causes of gynecologic cancer in women, a rare subtype, mucinous ovarian carcinoma (MOC), accounts for roughly 2%-3% of newly diagnosed cases. Ovarian carcinomas are classified into three broad subtypes: epithelial, germ, and stromal carcinomas. Of these, the largest category is epithelial ovarian carcinomas (EOCs), which contain low-grade and high-grade tumors such as high-grade serous carcinoma (HGSC), ovarian endometrioid carcinoma, clear cell carcinoma, and MOC. Among EOCs, HGSC is the most prevalent, making up 70% of EOCs, while MOC accounts for roughly 3%-4%, making it relatively rare [1]. Interestingly, research has shown MOCs to be distinct from EOCs in their histopathology, natural history, chemosensitivity, and prognosis [4,5].

The clinical presentation of our patient unveiled distinct characteristics, notably her age and postmenopausal status. Strikingly, the presence of an exceptionally large mass, measuring 33.5 cm in width, stood out, accompanied by a myriad of comorbidities attributable to its significant mass effect. On average, MOCs will present as a large, unilateral, multiloculated cystic mass, most often occurring in women between the ages of 20 and 40 years old [6,7]. Tumors can become large, with the mean size at presentation being 18 cm, often causing significant mass effects in the abdomen as a result. Tumor size has been suggested to indicate the primary source, with larger unilateral tumors favoring ovarian primaries. In a comprehensive retrospective series and analysis of the Surveillance, Epidemiology, and End Results (SEER) database, it was demonstrated that 79% of mucinous tumors are unilateral. When distinguishing between primary and metastatic mucinous carcinomas, primary MOCs displayed a lower tendency to bilateral involvement [8,9].

Patients who have advanced MOC generally exhibit poorer survival rates; this can be due to the overall aggressive nature of MOC, its tendency to metastasize, or its resistance to traditional platinum-based therapy, an approach that has been proven to be effective for other EOCs. Given similarities in pathological and molecular characteristics of both MOCs and gastrointestinal (GI) tumors, some retrospective studies suggest a benefit from empirically derived GI chemotherapeutic regimens. Only one trial, the Gynecologic Oncology Group trial 0241, compared capecitabine and oxaliplatin (a gastrointestinal chemotherapy regimen) with carboplatin and paclitaxel for MOC treatment. However, this was prematurely terminated due to the rarity of MOCs [7].

Most MOCs have a tendency to be sizable, and the preferred approach involves performing an exploratory laparotomy and removal of the affected adnexa. If the patient is postmenopausal, regardless of histology, a total hysterectomy combined with bilateral salpingo-oophorectomy can be considered as an option as was the case in this presentation [6]. The pathology of this case favored an ovarian primary based on the overall histologic and immunohistochemical findings; however, a gastrointestinal primary could not be entirely excluded. The specimen was categorized as FIGO stage IIB; this indicates tumor involvement of one or more ovaries with extension into the pelvic cavity and involvement of pelvic structures [10]. Further criteria for staging are available in the supplemental resources (Appendices). Theoretically, if the specimen had been determined to be a gastrointestinal primary, empirically derived gastrointestinal chemotherapy regimens could have been considered as one potential treatment. The decision to not pursue further surgical or chemotherapeutic treatment was based on the lack of malignant cells along with negative margins of resection in the specimen.

## Conclusions

Overall, MOC is a rare subset of ovarian cancers with distinct characteristics including its clinical presentation and tumor size, which underscores the need for careful consideration in diagnosis and treatment plans. MOC has potentially ambiguous origins, which can raise several challenges. However, advancements in understanding the pathology and treatment options for this disease offer a positive outlook for improved patient outcomes in the future. Ultimately, further research and clinical trials are essential to refine therapeutic approaches and enhance management strategies for patients with the disease.

## Figures and Tables

**Figure 4:**
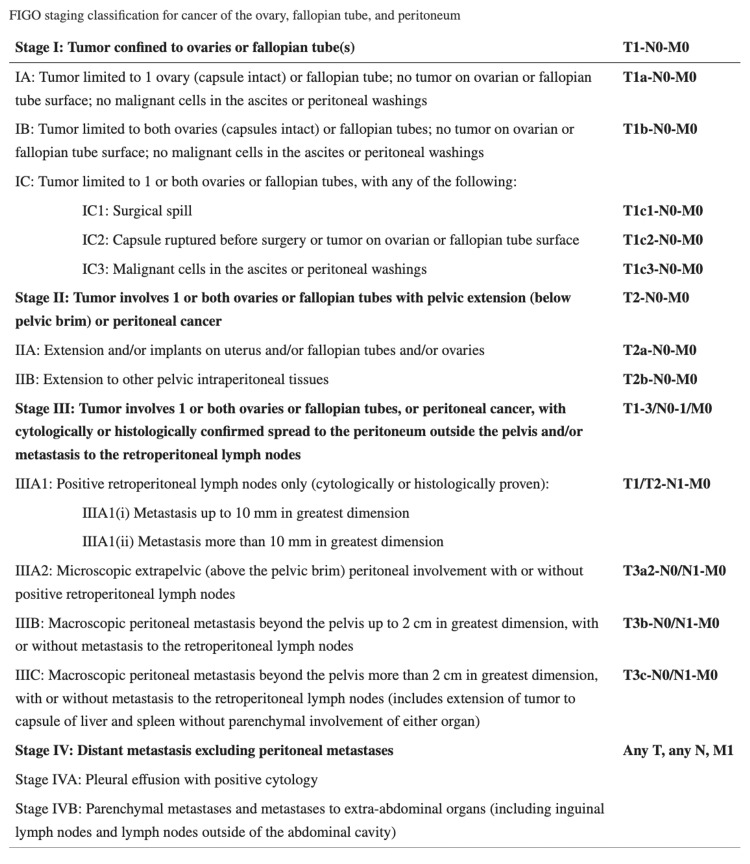
FIGO staging classification for cancer of the ovary, fallopian tube, and peritoneum. FIGO: International Federation of Gynecology and Obstetrics Source: Berek JS, Renz M, Kehoe S, Kumar L, Friedlander M: Cancer of the ovary, fallopian tube, and peritoneum: 2021 update. Int J Gynaecol Obstet. 2021, 155:61-85. 10.1002/ijgo.13878 [10]

## Appendices

Figure 4 presents the FIGO staging classification for cancer of the ovary, fallopian tube, and peritoneum.
